# Characterization of ESBL- and AmpC-Producing and Fluoroquinolone-Resistant *Enterobacteriaceae* Isolated from Mouflons (*Ovis orientalis musimon*) in Austria and Germany

**DOI:** 10.1371/journal.pone.0155786

**Published:** 2016-05-18

**Authors:** Igor Loncaric, Christoph Beiglböck, Andrea T. Feßler, Annika Posautz, Renate Rosengarten, Chris Walzer, Ralf Ehricht, Stefan Monecke, Stefan Schwarz, Joachim Spergser, Anna Kübber-Heiss

**Affiliations:** 1 Institute of Microbiology, University of Veterinary Medicine, Vienna, Austria; 2 Research Institute of Wildlife Ecology, University of Veterinary Medicine, Vienna, Austria; 3 Institute of Farm Animal Genetics, Friedrich-Loeffler-Institut (FLI), Neustadt-Mariensee, Germany; 4 Alere Technologies GmbH, Jena, Germany; 5 InfectoGnostics Forschungscampus, Jena, Germany; 6 Institute for Medical Microbiology and Hygiene, Technical University, Dresden, Germany; Institut National de la Recherche Agronomique, FRANCE

## Abstract

The aim of this study was to investigate the presence of β-lactamase producing or fluoroquinolone-resistant members of the family *Enterobacteriaceae* in European mouflons (*Ovis orientalis musimon*). The mouflon samples originated from nasal and perineal swabs and/or organ samples in cases of a suspected infection. Only one of the 32 mouflons was tested positive for the presence of *Enterobacteriaceae* that displayed either an ESBL/AmpC phenotype or were resistant to ciprofloxacin. The positively tested swab originated from a sample of the jejunal mucosa of a four-year old female mouflon. Two different colony morphotypes were identified as *Escherichia coli* and *Klebsiella pneumoniae*. These isolates were phenotypically and genotypically characterized in detail by a polyphasic approach. Both isolates were multi-drug resistant. The *E*. *coli* isolate belonged to the phylogenetic group B1 and sequence type (ST) 744 and harboured the β-lactamase genes *bla*_CTX-M-15_ and *bla*_OXA-1_. The *K*. *pneumoniae*, identified as ST11, harboured the β-lactamase genes *bla*_SHV-11_, *bla*_OXA-1_, and *bla*_DHA-1_ as well as the plasmid-mediated quinolone resistance (PMQR) gene *qnrB55*. The present study demonstrates that wild animals can acquire human-derived resistance determinants and such findings may indicate environmental pollution with resistance determinants from other sources.

## Introduction

*Enterobacteriaceae* producing β-lactamases are an emerging problem for human and animal health. The incidence of infections caused by extended spectrum β-lactamase (ESBL)- or plasmid-mediated AmpC β-lactamase-producing members of *Enterobacteriaceae* is increasing in humans [1]. Plasmid-borne ESBL and *ampC* genes can easily be transmitted by conjugation to other bacteria. In addition, various other resistance genes are frequently co-located on these plasmids and as a consequence are co-transmitted, limiting the therapeutic options in the case of an infection with these bacteria [2]. Due to proximity to humans, companion animals and livestock may act as a reservoir of bacterial pathogens producing ESBL and/or AmpC enzymes [3,4]. In recent years, wild animals became a subject of several studies addressing the isolation and characterization of β-lactamase-producing *Enterobacteriaceae*. Wild animals do not have necessarily direct contact with domestic animals and humans, but could become colonized or infected with antibiotic-resistant enterobacteria while sharing the same habitat or through global environmental pollution [5–8]. To determine the presence of antibiotic-resistant enterobacteria in Austrian wildlife, the Working Group for Clinical Microbiology and Animal Hygiene and the Research Institute of Wildlife Ecology started a pilot study in March 2012. The mouflon is a subspecies group of wild sheep. While scientific classification and designation is controversial, the mouflon is thought to be one of the two ancestors of all modern domestic sheep. The European mouflon (*Ovis orientalis musimon*) originates, most probably from feral domestic stock, introduced to the islands of Corsica and Sardinia in neolithic times [9]. Over the past thousands of years in the mountainous inner parts of these islands, this stock has given rise to the present-day subspecies. The horns of the mature rams, which weigh up to 50 kg, curl nearly a complete circle and are thus prized trophy animals by hunters. This has led to a widespread introduction of mouflons for hunting purposes into continental Europe including Austria. In Austria, mouflons are found and hunted mostly in the Alpine regions.

The aim of this study was to investigate mouflons submitted for necropsy at the Research Institute of Wildlife Ecology of the University of Veterinary Medicine, Vienna, Austria for the presence of ESBL-/AmpC-producing or fluoroquinolone-resistant members of the family *Enterobacteriaceae*.

## Materials and Methods

### Ethics statement

No specific permissions are required for this type of activity. All samples were from dead mouflons submitted explicitly for diagnostic pathological examinations in order to determine their respective cause of death. Pathological examination inherently incorporates bacteriological examinations of the gastrointestinal tract and other organs as appropriate. As the submitted animals are dead and the owners specifically requested the examinations, no further specific permissions are required. The examined species is a hunted game animal species in Germany and Austria and is neither endangered nor specifically protected in these two countries.

### Bacterial isolates

Since the beginning of the pilot study, 32 free-living mouflons were necropsied from March 2012 to January 2016 to determine the cause of death (Table 1). All mouflons sent to the Research Institute of Wildlife Ecology for postmortem analysis during the period of the study were included in this study. Different degrees of decomposition were no criteria for exclusion. During pathological examination, routine nasal and perineal swabs were taken and sent to the laboratory for bacteriological examination. Additionally, swab and/or organ samples were taken in cases of a suspected infection of the respective animal. Besides routine bacteriological examination, selective isolation of broad-spectrum cephalosporin-resistant *Enterobacteriaceae* and enterobacterial isolates with reduced susceptibility to fluoroquinolones was conducted. For this, each specimen was precultured at 37°C overnight in buffered peptone water (BPW) (Merck, Germany) supplemented with cefotaxime (1 mg/L) and then cultivated at 37°C overnight on MacConkey agar (MCA_CTX_) (Oxoid, Basingstoke, United Kingdom) supplemented with cefotaxime (1 mg/L). In parallel, each specimen was enriched at 37°C overnight in BD^™^ MacConkey Broth (BD, Heidelberg, Germany) and subcultivated on MacConkey agar supplemented with ciprofloxacin (0.06 mg/L) (MCA_CIP_) to isolate enterobacteria with reduced susceptibility to fluoroquinolones. After incubation on MCA_CTX_ and MCA_CIP_, one colony representing a distinct colony morphotype was regrown on MCA_CTX/CIP_ and on Mueller Hinton Agar II (BD, Heidelberg, Germany). For detection of members of the family *Enterobacteriaceae*, standard bacteriological techniques were used [10]. Enterobacterial isolates were rechecked for ESBL production by combination disk tests using cefotaxime and ceftazidime with and without clavulanic acid (Becton Dickinson, Heidelberg, Germany) according to the Clinical and Laboratory Standards Institute (CLSI) [11]. Furthermore, a cefoxitin (30 μg) and ciprofloxacin (5 μg) disk (BD, Heidelberg, Germany) were added to this test, to detect AmpC phenotypes as well as fluoroquinolone-resistant *Enterobacteriaceae*. ESBL as well as AmpC phenotypes were confirmed by MASTDISCS^™^ ID AmpC and ESβL test (Mast Diagnostics, Merseyside, UK).

**Table 1:** Overview of examined mouflons (*Ovis orientalis musimon*), year of examination, cause of death and locality. The mouflon, wherefrom ESBL- and AmpC-producing and fluoroquinolone-resistant *Enterobacteriaceae* were isolated, is marked with *.

Year	Cause of death	Locality
2012	Maedi-Visna Virus-associated pneumonia	Öttingen/Germany
2012	Lungworm-associated pneumonia; Hepatitis	Öttingen/Germany
2012	Lungworm-associated pneumonia	Öttingen/Germany
2012	Maedi-Visna Virus-associated pneumonia	Öttingen/Germany
2012	Bacteria and parasite associated pneumonia; Enteritis; Infection with Maedi-Visna Virus	Öttingen/Germany
2013	Infection with Maedi-Visna Virus	Öttingen/Germany
2013	Maedi-Visna Virus associated pneumonia	Öttingen/Germany
2013	Cachexia; Endoparasitosis; Enteritis	Vienna/Austria
2013	Endoparasitosis; Infection with lancet liver fluke	Vienna/Austria
2013	Endoparasitosis; Infection with lancet liver fluke	Vienna/Austria
2013	Cachexia; Endoparasitosis; Cirrhosis of liver	Miesenbach/Austria
2013*	Bacteria-associated gastroenteritis*	Orth an der Donau/Austria*
2013	Septicaemia; Bacteria and parasite associated enteritis	Rohr-Gebirge/Austria
2013	Chronic laminitis	Zell-See/Austria
2013	Cachexia; Enteritis; Infection with Maedi-Visna Virus	Kösingen/Germany
2013	Enteritis; Endoparasitosis; Infection with lancet liver fluke	Vienna/Austria
2013	Septicaemia; toxic nephrodegeneration	Schützen/Austria
2014	Endoparasitosis	Vienna/Austria
2014	Enteritis; Infection with lancet liver fluke	Vienna/Austria
2014	Enteritis; Endoparasitosis; Infection with lancet liver fluke	Vienna/Austria
2014	Maedi-Visna Virus-associated pneumonia and encephalitis	Rohr-Gebirge/Austria
2014	Chronic Hepatitis; Infection with lancet liver fluke	Vienna/Austria
2014	Maedi-Visna Virus-associated pneumonia and encephalitis	Miesenbach/Austria
2014	Septicaemia; Endoparasitosis; Old Trauma	Vienna/Austria
2015	Endoparasitosis; Ivy-Poisoning	Vienna/Austria
2015	Maedi-Visna Virus-associated pneumonia; Enteritis	Klein-Rust/Austria
2015	Septicaemia; Nephrosis; Infection with lancet liver fluke	Vienna/Austria
2015	Starvation	Vienna/Austria
2015	Gastroenteritis; Lungworm-associated pneumonia; Infection with lancet liver fluke	Vienna/Austria
2015	Enteritis	Vienna/Austria
2016	Pnemonia	Eggenburg/Austria

### Pathological changes of animals carrying ESBL, AmpC producing or fluoroquinolone resistant members of *Enterobacteriaceae*

All but one mouflon were negative for the presence of *Enterobacteriaceae* displaying either an ESBL or AmpC phenotype or were resistant to ciprofloxacin. The swab that yielded a positive result originated from a jejunal mucosa sample of an approximately four-year old female mouflon found dead in April 2013 in the Donau-Auen National Park, Orth an der Donau, Austria (48°09`N, 16°34`W; Table 1). The nutritional status was very good (27 kg) and the animal was pregnant with one foetus. Macroscopic findings included moderate hyperemia und emphysema of the lungs, moderate hyperemia and follicular swelling of the spleen, extensive hyperemia and swelling of the abomasal mucosa and moderate hyperaemia of the mucosa in duodenum and jejunum. The content of the small intestine was predominantly liquid and of a greyish-greenish colour. Histopathology revealed diffuse infiltration of the submucosa in the abomasum with mainly lymphocytes and macrophages. The sample yielded two different bacterial colony morphotypes which were identified as *E*. *coli* (isolate 928) or *Klebsiella* sp. (isolate 928k). The isolates were stored at -80°C for future use.

### Identification of bacterial isolates, antimicrobial susceptibility testing, and detection of resistance genes

Species characterization of both colony morphotypes was conducted as described elsewhere [10]. Antimicrobial susceptibility testing was performed by broth microdilution according to Clinical and Laboratory Standards Institute (CLSI) [11,12] for the following antimicrobial agents: ampicillin, amoxicillin/clavulanic acid, ceftiofur, cefquinome, cephalotin, cefotaxime, cefoperazone, imipenem, nalidixic acid, streptomycin, neomycin, gentamicin, ciprofloxacin, enrofloxacin, trimethoprim-sulfamethoxazole, tetracycline, doxycycline, chloramphenicol, florfenicol, and colistin. Preliminary screening for the presence of different *bla* genes was performed using different PCR approaches as described previously [10], and further investigated by miniaturized *E*. *coli* oligonucleotide arrays [13]. In the case of the *Klebsiella* isolate, the AMR-ve miniaturized array (Alere Technologies, Jena, Germany)(http://alere-technologies.com/en/products/lab-solutions/amr-ve-genotyping.html) was used. The *bla* gene as well as *qnr* families that responded positively in the array were further analysed by PCR and sequencing using primers as described elsewhere [10]. The nucleotide sequences obtained were compared to those registered in the GenBank and compared with published sequences in the NCBI database (http://www.ncbi.nlm.nih.gov/pathogens/beta-lactamase-data-resources/). Variable regions of class 1 and class 2 integrons were determined [10]. In addition, PCR amplification and DNA sequencing of the quinolone resistance-determining regions (QRDR) of *gyrA*, and *parC* were performed [14].

### Conjugation experiments and plasmid replicon typing

The transferability of *bla* genes by conjugation, characterization of transconjugants for their antimicrobial susceptibility, presence of the relevant *bla* genes, and co-transfer of other resistance genes was performed as described elsewhere [10]. The conjugative plasmids were subjected to PCR-based replicon typing (PBRT) as described previously [15].

### Molecular typing

The phylogenetic group of the *E*. *coli* isolate was determined [16]. Multilocus sequence typing (MLST) of *E*. *coli* isolates was performed as described previously [17]. Allelic profile and sequence type (ST) determinations were carried out according to the *E*. *coli* MLST database (http://mlst.warwick.ac.uk/mlst/dbs/Ecoli/). *K*. *pneumoniae* MLST was performed by using the primers described by Diancourt et al. [18]. Allelic profiles and sequence types (STs) were verified at the Institute Pasteur MLST database (http://bigsdb.web.pasteur.fr/klebsiella/klebsiella.html).

## Results

### Identification of bacteria and molecular characterisation

Using species-specific primers isolate 928 was confirmed as *E*. *coli*. The highest 16S rDNA gene sequence similarity observed for the isolate 928k (KU245543) was >99.7% with the type strains of the *K*. *pneumoniae* group [*K*. *pneumoniae* subsp. *ozaenae* (AF130982), *K*. *pneumoniae* subsp. *pneumoniae* (X87276), *K*. *pneumoniae* subsp. *rhinoscleromatis* (Y17657)]. The *E*. *coli* isolate belonged to the phylogenetic group B1 and to the multilocus sequence type (ST) 744, whereas the *K*. *pneumoniae* isolate was assigned to the ST11.

### Characterisation of antimicrobial resistance and conjugations experiments

The *E*. *coli* isolate 928 displayed an ESBL phenotype and was resistant to all antimicrobial agents tested except imipenem and colistin. *E*. *coli* isolate 928 carried the β-lactamase genes *bla*_CTX-M-15_ und *bla*_OXA-1_ and a variety of non-β-lactamase genes (Table 2). This isolate contained a class 1 integron with a variable part of ca. 1.7 kb in size which harboured an *aadA5* and a *dfr17* cassette. The transfer of the isolate 928 to the sodium azide-resistant *E*. *coli* J53 and sodium azide- and rifampicin-resistant *E*. *coli* MT 102 was demonstrated for both *bla* genes as well as for some non-β-lactamase genes (Table 2). Isolate 928 was positive for the FIA and FIB replicons. Array based geno-serotyping demonstrated that isolate 928 belonged to the O101:H09 serotype (Table 2). The following amino acid substitutions were detected in the QRDR regions of *gyrA* (83Ser→Leu, 87Asp→Asn) and *parC* (80Ser→Ile).

**Table 2:** Characterization of of ESBL—and AmpC producing and fluoroquinolone resistant *Enterobacteriaceae* isolated from mouflon.

Isolate	928	928k
Species	*E*. *coli*	*K*. *pnemoniae*
*bla* gene	*bla*_CTX-M-15_ (+), *bla*_OXA-1_	*bla*_SHV-11_, *bla*_OXA-1_, *bla*_DHA-1_
PG^a^	B1	n.a.
ST	ST744	ST11
Replicons	FIA, FIB	n.a.
Integron^b^	class 1 (ca. 1.7 kb)	−
Non-β-lactam resistance patternb	**AMP**, **AMC**, XNL, CEQ, CEP, FOT, FOP, CIP, ENRO, GEN, **SXT**, **TET**, DOX, CHL, FFN	AMP, AMC, CEP, FOP, CIP, ENRO, SXT, CHL, FFN
Non-β-lactam resistance genesb	*aac(6′)-IIc*, *aac(6′)-Ib*, ***aadA5*, *dfr17***, *catA1*, *catB3*, *mphA*, *mrx*, ***tet*(B), *sul1***	*aac(6′)-IIc*, *aac(6′)-Ib*, *aadA2*, *aphA*, *dfrA12*, *catA1*, *catB3*, *oqxAc*, *qnrB55*, *arr*, *sul1*
Mutation in *gyrA* and *parC*	*gyrA*: 83Ser→Leu, 87Asp→Asn	not detected
	*parC*: 80Ser→Ile	

^a^ Phylogenetic group

^b^ **bold** means positive also after testing of transconjugants. Abbreviations: AMP, ampicillin; AMC, amoxicillin/clavulanate; XLN, ceftiofur; CEQ, cefquinome; CEP, cefalotin; FOT, cefotaxime; FOP, cefoperazone; CIP, ciprofloxacin; ENRO, enrofloxacin; GEN, gentamicin; SXT, trimethoprim-sulfamethoxazole; TET, tetracycline; DOX, doxycycline; CHL, chloramphenicol; FFN, florfenicol.

^c^ encodes one component of an RND-family multidrug efflux pump, OqxAB.

The *K*. *pneumoniae* isolate 928k displayed both, an ESBL and an AmpC phenotype and was susceptible to imipenem, gentamicin, streptomycin, tetracyclines, and colistin. It carried the β-lactamase genes *bla*_SHV-11_, *bla*_OXA-1_, and *bla*_DHA-1_ as well as several non-β-lactamase genes including the *qnrB55* gene. However, none of the resistance genes could be transferred to the recipient strains (Table 2). Moreover, no mutations were seen in the QRDR regions of the *gyrA* and *parC* genes.

## Discussion

So far, information regarding physiological and pathogenic bacterial microbiota from mouflons is scarce. In the present study, the phenotypic and genotypic characteristics of β-lactamase-producing *Enterobacteriaceae* isolates from a mouflon were investigated. Out of the 32 mouflons included in this study, only one animal was positive for such isolates and an *E*. *coli* ST744 of phylogroup B1 with an ESBL type (*bla*_CTX-M-15_, *bla*_OXA-1_) as well as a *K*. *pneumoniae* ST11 with an ESBL type and AmpC type (*bla*_SHV-11_, *bla*_OXA-1_, *bla*_DHA-1_), which also carried the PMQR gene *qnrB55*, were identified from the same sample. Phenotypic and genotypic tests confirmed that both isolates fulfilled the requirements to be considered as multi-drug resistant [19].

*E*. *coli* belonging to ST744 and carrying genes for ESBL- or AmpC-β-lactamases have been isolated from humans, companion and wild animals [7,10,20–23]. Regarding wildlife, *E*. *coli* ST744 isolates were isolated in Germany from black kites (*Milvus migrans*) and from a buzzard (*Buteo buteo*) and were characterized as phylogroup A-ST744-*bla*_CTX-M-1_-*bla*_TEM-1like_ [8]. Recently, our working group described the frequent presence of ESBL and AmpC producing *Enterobacteriaceae* isolated from rooks (*Corvus frugilegus*) in Austria [10]. In that study, three *E*. *coli* isolates belonging to ST744 were detected, i.e., A-ST744-*bla*_CTX-M-1_, A-ST744-*bla*_CTX-M-3_, and C-ST744-*bla*_CTX-M-1_. *K*. *pneumoniae* ST11 is an epidemic clone that has been isolated from humans worldwide, and it is associated with the spread of carbapenemase genes such as *bla*_OXA-48_,*bla*_NDM_, and *bla*_KPC-2_ as well as aminoglycoside resistance mediating 16S rRNA methylase genes such as *rmtB* [24–27]. This clone carrying different *bla* genes has also been reported in association with clinical signs in companion animals [28–30]. To the best of our knowledge, there is only one report regarding the association of *K*. *pneumoniae* ST11 carrying *bla* genes with wildlife [31]. In contrast to the studies dealing with the presence of *bla* genes and PMQR in *E*. *coli* from wildlife, information about other members of *Enterobacteriaceae* including *Klebsiella* spp. harbouring these genes are scarce. Recently, Janatova et al. [32] reported the presence of antimicrobial-resistant *Enterobacteriaceae* from humans and wildlife in Dzanga-Sangha Protected Area, Central African Republic. Among other bacteria, PMQR-positive *K*. *pneumoniae* were obtained from African buffalo (*Syncerus caffer*), Peters’ duiker (*Cephalophus callipygus*), unhabituated gorilla (*Gorilla gorilla gorilla*) and *K*. *pneumoniae* carrying *bla*_TEM-1_ was isolated from unhabituated gorilla. Albrechtova et al. [33] isolated a PMQR (*qnrB13*, *oqxA*)-positive *K*. *pneumoniae* isolate from a western chimpanzee (*Pan troglodytes verus*). β-Lactamase-producing *E*. *coli* (*bla*_CTX-M-14_ or *bla*_TEM-19_) and *K*. *pneumoniae* (*bla*_CTX-M-15_, *bla*_SHV-12_, or *bla*_SHV-102_) were isolated also from glaucous gulls (*Larus hyperboreus*) in Alaska, USA [34]. Very recently, the occurrence of ESBL-producing bacteria was detected in fecal samples from herring gulls (*Larus argentatus*) and lesser-black backed gulls *(Larus fuscus*) in northern Europe, and yellow-legged gulls (*Larus michaelis*) in southern Europe with with *bla*_CTX-M-1_ and *bla*_CTX-M-14_ as the most predominant variants [35].

It is not possible to determine in retrospect when and under which conditions the mouflon from this study has acquired the multi-resistant *E*. *coli* and *K*. *pneumoniae* isolates. Since the free-living mouflons have not been treated with cephalosporins or fluoroquinolones, it appears as if there is no obvious direct selective pressure under which such strains may have been acquired. More likely is that such multi-resistant *Enterobacteriaceae* have been taken up via environmental contamination. The affected mouflon in the present study was found close to the study site and almost at the same time, where different multi-drug resistant *Enterobacteriaceae* were isolated from rooks [10]. As such, multi-resistant *Enterobacteriaceae* from rooks’ droppings may have resulted in an environmental contamination of that area. Further studies have shown that the *bla*_CTX-M-15_ gene is one of the most common types of ESBL genes worldwide and it is the most prevalent ESBL gene in humans [36]. Thus, the presence of *E*. *coli* harboring this gene in a mouflon could also be the result of a human-to-animal transfer or a human-to-environment contamination with subsequent uptake of the strain by the mouflon. The affected mouflon was found in a national park, located between the European capitals Vienna and Bratislava, which is frequently visited by humans (who are also allowed to be accompanied by their dogs). Thus, the potential for transmission of resistant *Enterobacteriaceae* between visitors, companion and wild animals—including the contamination of the environment by human or companion animal excretions that contain multi-resistant *Enterobacteriaceae*–cannot be excluded. Whether or not the finding of ESBL-/AmpC-producing and PMQR gene-carrying *Enterobacteriaceae* in a single mouflon represents a potential public health risk remains debatable. However, the finding of such multi-resistant isolates in a wildlife animal, which was not subjected to antimicrobial therapy, underlines the connection between human medicine, veterinary medicine and the environment as documented in the One Health principle (http://www.onehealthinitiative.com/). Therefore, the finding of such resistant bacteria might be used as an indicator for environmental pollution.
